# Retrospective review of bacteriological profiles and antibiogram in a tertiary neonatal unit

**DOI:** 10.4102/sajid.v38i1.537

**Published:** 2023-12-08

**Authors:** Philile F. Buthelezi, Fathima Naby, Yashodhara Kannigan

**Affiliations:** 1 Department of Paediatrics and Child Health, Nelson R Mandela School of Medicine, Faculty of Health Sciences, University of KwaZulu-Natal, Pietermaritzburg, South Africa

**Keywords:** neonatal sepsis, neonatal mortality, antibiotic susceptibility, antibiotic resistance, empirical antibiotics

## Abstract

**Background:**

Neonatal sepsis remains a major cause of morbidity and mortality. Therefore, early detection and initiation of appropriate empirical antibiotic therapy are crucial.

**Objectives:**

The aim of this study was to describe the antibiogram of the neonatal intensive care unit at Grey’s Hospital, a tertiary hospital in KwaZulu-Natal.

**Method:**

This was a retrospective descriptive study, reviewing positive cultures from Grey’s Hospital tertiary neonatal intensive care unit (NICU) in KwaZulu-Natal, South Africa for a 3-year period (01 January 2017 to 31 December 2019). All positive cultures from all sites were included.

**Results:**

There were 1314 positive organisms cultured. Late-onset sepsis (89.3%) predominated over early-onset sepsis (10.7%). Blood was the source for 55.2% (725/1314) of positive cultures. Of the 1314 organisms cultured, 53.7% (706/1314) were Gram-positive, 45.7% (601/1314) were Gram-negative and 0.5% (7/1314) were Candida species. *Klebsiella pneumoniae*, 23.5% (313/1314) was the most frequent Gram-negative organism. It was noted to have high resistance to the unit’s first-line antibiotic regimens; 99% were resistant to ampicillin and 92% resistant to gentamicin.

**Conclusion:**

Blood cultures yielded most positive results with a predominance of Gram-positive organisms and late-onset sepsis. A significant proportion of the cultured organisms were resistant to the first-line antimicrobials utilised in the unit, ampicillin and gentamicin.

**Contribution:**

Ongoing surveillance on positive cultures is recommended to assess the effectiveness of the unit’s current empirical antimicrobial guideline.

## Introduction

Globally, sepsis remains a major cause of morbidity and mortality, with limited mortality reduction progress despite recent advances in neonatal care.^1^ According to the World Health Organization, about 4 million babies die in their first 28 days of life. Infection is the leading cause of newborn death, accounting for 36% of all deaths.^1,2^ In South Africa, neonatal sepsis is the third most common cause of neonatal death.^3^

Sepsis from severe bacterial infections remains the leading cause of mortality and morbidity in sub-Saharan Africa.^4,5^ South African (SA) data have reported the incidence of neonatal sepsis to be 8.5% – 10.0%, with late-onset sepsis (LOS) accounting for 83.2% – 94.3% of these infections.^6^ In developing countries, neonatal sepsis causes 1.6 million deaths per year.^7^ In sub-Saharan Africa, sepsis-related neonatal mortality rates range between 17.0% to 29.0%.^3^ In South Africa, the mortality rate varied between 24.2% to 40% and 19.7% to 22.5% for early-onset sepsis (EOS) and LOS, respectively.^6^

Locally in KwaZulu-Natal, neonatal sepsis caused 11.6% of neonatal deaths.^2^ Most of deaths are because of Gram-negative sepsis (69.2% – 80%). An estimated 31.0% of deaths from neonatal sepsis are related to antimicrobial resistance.^6^ Early-onset sepsis has been on the decline in epidemiological research, while LOS has been on the rise.^8^

Sepsis is the body’s systemic immunological response to an infectious process that can lead to end-stage organ dysfunction and death. Neonatal sepsis presents with non-specific symptoms and signs of infection in the first 28 days of life.^3,9^ Both term and premature neonates have undeveloped immune systems and are at significant risk of sepsis because the immune system plays a major role in fighting infections.^10^ Early-onset sepsis presents in the first 72 h of life and LOS presents after 72 h of life.^1^

Early-onset neonatal sepsis is usually through vertical transmission with maternal chorioamnionitis and maternal group B *Streptococcus* (GBS) colonisation being well-recognised risk factors.^11,12^ In South Africa, the most common causes of early neonatal sepsis were the Gram-negative organisms, *E. coli, Klebsiella pneumoniae* and GBS, most common organisms in the developing and the developed world.^3,6^ The prevalence of GBS has decreased since the advent of prenatal screening and intrapartum treatment.^8,13^

The most prevalent organisms from a recent study by Velaphi et al. on the aetiology and incidence of EOS was GBS.^14^ *Listeria monocytogenes, Staphylococcus aureus, Enterococcus faecium, Enterococcus faecalis* and Gram-negative bacteria. In rare cases, EOS can be caused by *Streptococcus pneumoniae, Streptococcus pyogenes, Haemophilus influenzae*, the viridans streptococci and *Pseudomonas aeruginosa*.^6^

Late-onset neonatal sepsis is the result of horizontal transmission from the hospital environment including in-patient and caregivers.^15,16^ The incidence of LOS has decreased as a result of advancement in treatments that improve the survival of premature infants.^8^ The development of LOS is inversely related to gestational age and birth weight.^8^

The microbiological characteristics of LOS have changed worldwide.^8^ In South Africa, predominant organisms causing late-onset neonatal sepsis are Gram-negative organisms, namely *Acinectobacter* species, *Klebsiella* species, *Enterobacter* species and *E. coli*.^7^ Coagulase-negative staphylococcal infection has become the most common cause of LOS in developed countries. These account for 53.2% to 77.9% of LOS, while in some low- and middle-income regions, it contributes for 35.5% to 47.4%.^8^

Fungal infections are being recognised as a cause for late-onset sepsis.^3^ Candida species one of the most common causes of fungal sepsis in extremely low birthweight neonates. In South Africa, neonatal candidaemia is commonly caused by *Candida parapsilosis*.^3,6^

Culture remains the gold standard for the diagnosis of neonatal sepsis.^1,6,17^ Cultures should be done before initiating antibiotics.^18^ Prior maternal antibiotic treatment, as well as intermittent or low-density bacteraemia in newborns, can result in negative culture results.^18^ Cerebrospinal fluid (CSF) culture should be performed in all neonates with suspected sepsis, as more than a quarter of neonates with blood culture-positive sepsis have meningitis.^19^

Antimicrobials are essential for the treatment of neonatal sepsis.^20^ Antibiotics are the most often used antimicrobials in neonatal intensive care units (NICUs), with antibiotics being administered in up to 72% of NICU patients in general and 85% of very low birth weight infants in particular.^21^

A combination of an aminoglycoside and either benzylpenicillin or ampicillin is the most common empiric antibiotic regimen for EOS in many countries. This combination is supported by both the National Institute for Health and Clinical Excellence (NICE) and American Academy of Paediatrics (AAP) guidelines.^22^

When using antimicrobials, one must be very cautious of antimicrobial resistance.^4^ This is a growing public health emergency globally and poses a threat to our health systems because infections from resistant microbes are more difficult and costly to treat and are associated with increase in morbidity and mortality.^3,23^

A multidrug-resistant (MDR) microorganism is defined as one not susceptible to at least one drug in two or more antimicrobial classes tested.^24^ Studies in South Africa have documented the emergence of drug resistance to multiple antibiotics in NICUs.^25,26^ Knowing local antibiograms improves clinicians’ prescribing behaviour thereby reducing the risk of antibiotic resistance.

Our study aims to describe the antibiogram of the NICU at Grey’s Hospital, a tertiary hospital in KwaZulu-Natal. This will assist doctors in prescribing appropriate antibiotic regimens for patients admitted to the facility. Furthermore, this study will contribute towards the formulation of local empirical antibiotic guidelines and will illustrate an approach that can be adopted by other facilities.

## Methods

### Study design

This was a retrospective descriptive study, reviewing the positive cultures from Grey’s Hospital tertiary NICU in KwaZulu-Natal, South Africa over a 3-year period (01 January 2017 to 31 December 2019). Culture sites included blood, endotracheal aspirations (ETAs), superficial pus swab, urine, CSF, gastric washing, arterial and venous catheter tip and fluid aspirates.

### Study site

The NICU at Grey’s Hospital is a 26 bedded tertiary unit (6 intensive care beds and 20 high care beds) in Pietermaritzburg, KwaZulu-Natal midlands and falls under the uMgungundlovu health district umbrella. The unit admits patients born in the facility itself as well as referrals from the northwestern KwaZulu-Natal providing service to 14 health districts and three regional hospitals for both medical and surgical patients.

### Study population

All positive cultures and their sensitivity results from the patients admitted or transferred into Grey’s NICU over a 3-year period (01 January 2017 until 31 December 2019) were included. Positive cultures were extracted from the National Health Laboratory Service (NHLS) computerised database. Information on patient name, date of birth, dates when cultures were taken, site of culture, organisms identified and antibiotic susceptibilities were collected. Data on date of admission, date of discharge, date of death, diagnosis, birthweight or current weight was obtained from discharge summaries and in-patient notes.

### Study definition

Sepsis was classified as early-onset (< 3 days old) and late-onset sepsis (≥ 3 days old) measured from the date of birth until collection of the positive culture.

### Laboratory methods and information handling

In patients with suspected sepsis, culture samples were collected in Grey’s NICU ward and transported to the NHLS Microbiology Laboratory for incubation and susceptibility testing. They were then processed and prepared for Gram stain and agar plates. The laboratory uses the VITEK system (BioMerieux, France) for automated microbial identification and susceptibility testing. For colistin susceptibility, broth microdilution method was performed.

Microsoft Excel was used to enter all the data. Statistical Package for Social Sciences (SPSS) software was used to conduct the analyses (version 17). To describe the data, descriptive statistics, frequencies and percentages were calculated.

### Ethical considerations

Permission to conduct the study was granted by the Biomedical Research Ethics Committee (BREC) of the University of KwaZulu-Natal (BREC/00002531/2021). Grey’s Hospital management, KwaZulu-Natal Department of Health (ref KZ 202103 022) and the NHLS head of department, PR2113722, all granted authorisation for the research location to be used for the study.

## Results

Over the 3-year period, from January 2017 to December 2019, 1314 organisms were cultured from 571 patients. The maximum positive cultures per patient was 16.

Out of 571 patients, 82 had missing clinical information. Of the positive cultures, 66.7% (326/489) were patients with medical conditions and 33.3% (163/489) were patients with surgical conditions. Thirty-two percent (156/489) of patients with positive cultures were premature neonates.

**FIGURE 1:**
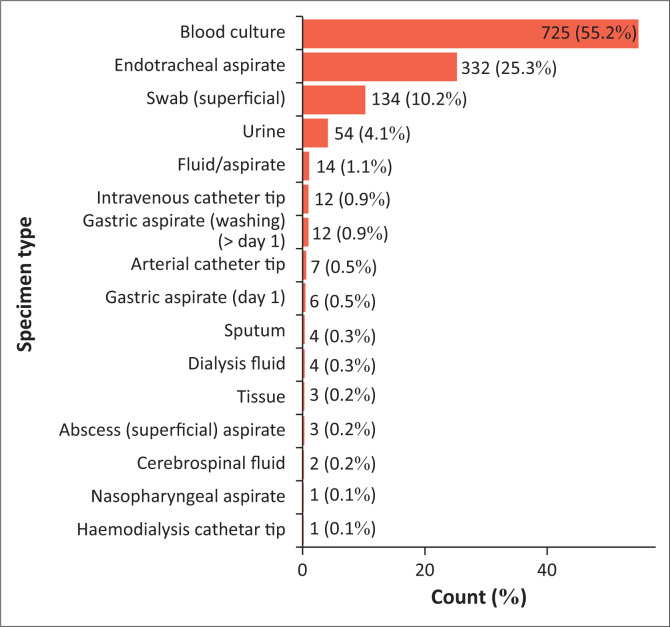
Specimen type (*n* = 1314).

As noted in Figure 1, of the total number of positive cultures, blood accounted for 55.2% (725/1314) of positive cultures followed by ETA specimens 25.3%, superficial pus swab 10.2% and urine specimens 4.1%.

Gram-positive bacteria accounted for 53.73% (706/1314) of the positive cultures. Gram-negative bacteria and fungi contributed 45.74% (601/1314) and 0.53% (7/1314), respectively.

In this study, 89.3% (1173/1314) of the organisms isolated occurred during LOS and 10.7% (141/1314) during EOS. *Klebsiella pneumoniae* accounted for 15.6% (22/141) of EOS and 24.8% (291/1173) of LOS. Other organisms cultured during EOS were *Staphylococcus* species 77/141 (54.6%), *E. coli* 7/141 (4.9%), *Streptococcus* species 7/141 (4.9%) and *Enterobacter cloacae* complex 6/141 (4.3%). Other organisms that contributed to LOS were *Staphylococcus* species 537/1173 (45.7%), *Acinetobacter* species 91/1173, (7.8%), *Enterococcus* species 57/1173 (4.9%) and *E. cloacae* complex 48/1173 (4.1%) (see Table 1).

**TABLE 1:** Early-onset versus late-onset sepsis.

Organisms subtype	EOS < 3 days old (*n* = 141, 10.7%)		LOS ≥ 3 days old (*n* = 1173, 89.3%)	
	*n*	%	*n*	%
Gram-negative organisms (*n* = 601)	52	-	549	-
*Klebsiella pneumoniae subsp pneumoniae*	22	42.3	291	53.0
*Acinetobacter complex*	5	9.6	91	16.6
*Enterobacter cloacae complex*	6	11.5	48	8.7
*Serratia marcescens*	1	1.9	27	4.9
*Escherichia coli*	7	13.5	24	4.4
*Stenotrophomonas maltophilia*	2	3.8	24	4.4
*Pseudomonas aeruginosa*	4	7.7	13	2.4
*Klebsiella oxytoca*	1	1.9	8	1.5
*Sphingomonas paucimobilis*	1	1.9	3	0.5
*Burkholderia cepacia*	0	0.0	6	1.1
*Enterobacter aerogenes*	0	0.0	3	0.5
*Chryseobacterium indologenes*	1	1.9	3	0.5
*Citrobacter freundii*	1	1.9	2	0.4
*Proteus mirabilis*	0	0.0	2	0.4
*Pantoea agglomerans*	1	1.9	1	0.2
*Providencia rettgeri*	0	0.0	1	0.2
*Raoultella ornithinolytica*	0	0.0	1	0.2
*Morganella morganii subsp morganii*	0	0.0	1	0.2
Gram-positive (*n* = 706)	88	-	618	-
*Staphylococcus epidermidis*	47	53.4	347	56.1
*Staphylococcus species*	25	28.4	112	18.1
*Staphylococcus aureus*	5	5.7	78	12.6
*Enterococcus faecium*	1	1.1	32	5.2
*Enterococcus faecalis*	2	2.3	25	4.0
*Streptococcus species*	7	8.0	22	3.6
*Bacillus species*	0	0.0	1	0.2
*Enterococcus species*	0	0.0	1	0.2
*Aerococcus viridans*	1	1.1	0	0.0
Fungal (*n* = 7)	1	-	6	-
*Candida albicans*	1	100.0	4	66.7
*Candida krusei*	0	0.0	1	16.7
*Candida parapsilosis*	0	0.0	1	16.7

The top three Gram-negative organisms cultured were *K. pneumoniae* 23.5% (313/1314), *Acinetobacter baumanii* 90/1314 and *E. cloacae* 52/1314.

*Candida albicans* species was identified in five samples of the seven fungal infections cultured. Sixty percent (3/5) were cultured from blood, with all sensitive to fluconazole (100%).

Of all positive blood cultures, the most common organisms cultured were *Staphylococcus* species 51.6% (374/725), *Klebsiella* species 14.6% (106/725) and *Acinetobacter* species 1.9% (14/725).

Twenty-five percent (332/1314) of all positive cultures were from ETA. Organisms cultured were *Klebsiella* species 33.4% (111/332), followed by *Acinetobacter* species at 18.1% (60/332) and *Enterobacter* species 6.3% (21/332). *Staphylococcus* species only accounted for 4.5% of positive cultures from endotracheal aspirates.

The percentage of positive urine cultures was 4.1% (54/1314) of all positive cultures, with 64.1% (35/54) of those cultures being *Klebsiella* species and 18.1% (10/54) being *Enterobacter* species.

In total, 313 isolates of *K. pneumoniae* were isolated (Table 2): 106 (34%) from blood cultures, 111 (35%) from ETAs, 35 (11%) from urine and the remaining 20% from other sites. Gentamicin, amoxicillin-clavulanate and piperacillin-tazobactam each had 8%, 11% and 37% antibiotic susceptibility rates, respectively. Carbapenem resistance was detected in 52 (17%) of *Klebsiella* species. Ampicillin resistance was 99.4%. Resistance rates observed for the beta-lactam agents, amoxicillin-clavulanate and piperacillin-tazobactam, were 51% and 41%, respectively. Gentamicin resistance occurred in 92% and ciprofloxacin resistance in 52%. Intermediate sensitivity was noted in 38% of amoxicillin-clavulanate and 22% of piperacillin-tazobactam.

**TABLE 2:** Percentage resistance of *Klebsiella pneumoniae, Acinetobacter* species and *Enterobacter cloacae* to first line, second line, third line and forth line antibiotics.

Antimicrobials	*Klebsiella pneumoniae* (*n* = 313)		Acinetobacter species (*n* = 96)		*Enterobacter cloacae* (*n* = 54)	
	*n*	%	*n*	%	*n*	%
Ampicillin	311	99	15	16	42	78
Gentamycin	287	92	78	81	42	78
Piperacillin tazobactam	128	41	87	87	23	43
Amikacin	44	14	68	71	3	6
Meropenem	52	17	85	89	4	7
Colistin	0	0	0	0	0	0

Of the 96 isolates of the *Acinetobacter* species (Table 2), 64/96 (67%) were cultured from ETA, followed by 15/96 (16%) from blood, 2/96 (2%) from urine and 15/96 (16%) from other sites. Colistin had an antibiotic susceptibility rate of 89%, while meropenem had a susceptibility rate of 3%.

*Enterobacter cloacae* made up 54 (Table 2) of the 1314 cultured organisms, with 6/54 (11%) isolated from blood cultures, 21/54 (39%) from ETA, 7/54 (13%) from urine and 20/54 (37%) from other sites. Gentamicin and piperacillin-tazobactam had an antibiotic susceptibility rate of 17% and 30%, respectively. Four (7%) of the identified *Enterobacter* species were carbapenem resistant. Resistance to piperacillin-tazobactam was 43% and ampicillin was 78%.

## Discussion

In the current study, we describe the positive cultures seen in a tertiary neonatal facility in KwaZulu-Natal. We found more cultures positive in medical than surgical patients. Premature neonates with low birth weight had the highest rates of positive cultures. Blood culture was the most frequent culture site of positive cultures.

Early-onset sepsis has been on the decline, while LOS has been on the rise.^9,26^ Dong et al. reviewed LOS and reported that with advances in neonatal care medicine, there has been increasing survival of neonates and increased incidence of LOS.^9^ Late-onset sepsis predominated over early-onset sepsis in this review, which is comparable to studies from South African tertiary level neonatal units where LOS accounted for 83.7%, 86.8% and 85.7% by Lebea et al., Pillay et al. and Van Staaden et al., respectively.^1,3,8^

*Escherichia coli, K. pneumoniae* and GBS are the most common pathogens causing early neonatal sepsis in South Africa, as well as in other developing and developed countries.^27^ The prevalence of GBS has decreased after the introduction of prenatal screening and intrapartum antibiotics.^7,8^ In our review, *E. coli* and *K. pneumoniae* were the most significant organisms causing EOS. Group B Streptococcus was only cultured once.

In this review, significant organisms in both LOS and EOS included *K. pneumoniae* and *Staphylococcus* species. This is consistent with other studies in South Africa.^1,3,7^ *Staphylococcus* species have become the most common cause of LOS in developed countries.^8^ According to Lebea et al., coagulase-negative staphylococci are one of the major causes of newborn sepsis.^7^

*Enterococcus* species were identified by Dramowski et al. as a major contributor to gram-positive sepsis in neonates.^24^ In the current study, there was a predominance of *E. faecium* and *E. faecalis* in LOS.

Gram-positive organisms were the most prevalent organisms, with *Staphylococcus epidermidis* identified as the most common organism. These results are similar to reports from India and other African nations.^3,4^ This needs to be interpreted carefully as this organism is often regarded as a contaminant. To assist with decisions regarding the clinical relevance, clinical correlation is necessary.

Poor blood culture sampling technique may be the cause of the rise in *Staphylococcus* species. However, it may be attributed to the neonates in NICUs having immature immune systems with foreign bodies such as central venous catheters, placing them at increased risk for infections.^7,11^ To improve the quality of samples taken, the healthcare team members must be educated on the proper blood culture collection techniques.^22^

Research from other low- and middle-income countries has also shown that after controlling contamination, *Staphylococcus epidermidis* was a major pathogen of newborn sepsis.^1,3,7^ Determining clinical significance in this study was difficult because of the lack of clinical data. As this study is based on laboratory surveillance, all clinical information was not gathered. Despite the evidence supporting *Staphylococcus epidermidis* as a pathogen of neonatal sepsis, isolates may still indicate contamination.^4^

In South Africa, the most prevalent Gram-negative organisms causing neonatal sepsis are *Acinectobacter* species, *Klebsiella* species, *Enterobacter* species and *E. coli*.^6^ These findings are similar to our review as the most common Gram-negative organisms were *K. pneumoniae, Acinetobacter* and *E. cloacae*.

Fungal infection rates were low. This is an unexpected finding as premature and low birth weight neonates are highly susceptible to fungal infection.^3^ The cultured fungal organisms were *Candida albicans, Candida krusei* and *C. parapsilosis. Candida albicans* was the predominant cause of neonatal candidemia, in contrast to other South African studies where *C. parapsilosis* was the most common candida species.^1,3^

Resistant organisms are becoming more prevalent in NICUs.^3,25^ This is a growing public health emergency globally and is associated with increase in morbidity and mortality.^3,22^ Gram-positive and Gram-negative bacteria showed poor susceptibility to first- and second-line antibiotics in our review. However, susceptibility to broad-spectrum antibiotics such as carbapenems was maintained.

Similar findings were noted in KZN where a reduction in susceptibility to first- and second-line antibiotics, to Gram-positive, Gram-negative organisms and fungi was also demonstrated.^3^ However, susceptibility to broad-spectrum antibiotics such as vancomycin for Gram-positive organisms and carbapenems for Enterobacterales was maintained.^3^ The high number of antimicrobial-resistant isolates suggests an infection control problem in the unit. This was previously noted, and infection prevention and control measures were adopted, which included audits on hand washing and antimicrobial stewardship rounds with the in-house paediatric infectious disease team.

Fluconazole susceptibility patterns in neonatal units are variable and based on the species of the predominating fungal pathogen.^4^ The practice in NICUs of giving fluconazole prophylaxis to high-risk patients may select fluconazole-resistant species.^5^ All candida isolates in this study were sensitive to fluconazole unlike other South African studies that reported fluconazole resistance, more frequently among *C. parapsilosis*.^3,4^

Reduced ampicillin susceptibility was noted, as in other African studies.^5,6^

The NICE and the AAP guidelines both support the combination of an aminoglycoside and either benzylpenicillin or ampicillin as first-line antibiotic therapy for neonatal sepsis.^6^ Choosing an appropriate empiric antimicrobial regimen at Grey’s NICU remains a challenge.

In view of the high levels of antimicrobial resistance observed, ampicillin and piperacillin-tazobactam as first and second-line agents, currently used in this NICU, are inappropriate. Meropenem provided optimal empiric cover in this study.

This study was conducted at a single centre, and therefore results cannot be generalised. Within the study population, premature neonates were not stratified and might have a different culture result from other neonatal groups. Limited information was available, including some of the clinical data; therefore, the ability of this study to identify clinically significant staphylococcus infection was inadequate.

## Conclusion

The majority of the organisms that were cultured were resistant to first- and second-line antimicrobials, ampicillin and gentamicin, but had good susceptibility to meropenem. Therefore, ongoing surveillance on positive cultures is recommended to assess the effectiveness of the unit’s current empirical antimicrobial guideline.
